# Evaluation of the effect of apixaban using a viscoelastic coagulation assay with Russell’s viper venom reagent

**DOI:** 10.1186/s40981-021-00445-9

**Published:** 2021-05-06

**Authors:** Kaoru Suzuki, Nobuyuki Katori, Yoshihiro Kimura, Takako Terui, Hiroshi Sunaga, Shunsuke Kobayashi, Shoichi Uezono

**Affiliations:** 1 Department of Anesthesiology, The Jikei University School of Medicine, 3-25-8 Nishishinbashi, Minato-ku, Tokyo, 105-8461 Japan; 2 Department of Orthopedics, The Jikei University School of Medicine, 3-25-8 Nishishinbashi, Minato-ku, Tokyo, 105-8461 Japan

**Keywords:** Anticoagulation, Anti-Xa, Apixaban, Russell’s viper venom

## Abstract

**Background:**

Conventional coagulation tests, such as prothrombin time and activated partial thromboplastin time, are not sensitive to anticoagulation by apixaban. We evaluated the antithrombotic effect of apixaban using a Russell viper venom (RVV) test for a patient who underwent posterior spine fusion surgery.

**Case presentation:**

An 84-year-old man was scheduled for percutaneous posterior spine fusion. He continued apixaban until the night before surgery and resumed it on the first day after surgery. We performed an RVV test as point-of-care coagulation monitoring in combination with chromogenic anti-activated factor X (anti-Xa) activity, prothrombin time, and activated partial thromboplastin time. Clotting time with the RVV test was prolonged according to the anti-Xa activity of apixaban, which was in the therapeutic range during surgery.

**Conclusions:**

An RVV test might be useful as a point-of-care assay for estimation of the anti-Xa level induced by apixaban during the perioperative period.

## Background

Conventional coagulation tests, such as prothrombin time (PT) and activated partial thromboplastin time (APTT), for monitoring of the anticoagulant effect of direct oral anticoagulants are not always required. However, there are circumstances, including emergency surgery, trauma, life-threating bleeding, acute kidney injury, etc., in which assessment of anticoagulant effect would be desirable. Although PT and APTT are the conventional coagulation tests used for the monitoring of anticoagulant drugs, such as warfarin and heparin, these tests are not sensitive to direct oral anti-activated factor X (anti-Xa) drugs such as apixaban [1].

Recently, a point-of-care, whole-blood, and viscoelastic device that uses active chip technology (ClotPro; enicor GmbH, Munich, Germany) has been approved for clinical use. In addition to established thromboelastometry assays, ClotPro provides a unique assay that can evaluate the inhibitory effect on Xa using Russell’s viper venom (RVV), which has high sensitivity to anti-Xa drugs. We evaluated the antithrombotic effect of apixaban using ClotPro and RRV for a patient who underwent posterior spine fusion surgery.

## Case presentation

The patient was an 84-year-old man with a height of 176 cm and a body weight of 80 kg who was scheduled for minimally invasive posterior spine fusion (T9–L3) for the treatment of a lumbar compression fracture. He had a history of hypertension, atrial fibrillation, and chronic kidney disease and was taking amlodipine and apixaban (5 mg twice daily). Preoperative serum examination indicated moderate loss of kidney function, which was confirmed by a serum creatinine level of 1.05 mg/dL and an estimated glomerular filtration rate of 52 mL/min/1.73 m^2^. Preoperative coagulation tests indicated prolonged values for both APTT and PT-international normalized ratio (40.1 s and 1.7, respectively). Although temporary interruption of apixaban is commonly recommended for the perioperative period (usually 48–72 h before surgical procedures, such as spine surgery, with high bleeding risk) [2], the patient continued apixaban until the night before surgery, according to the instruction of his cardiologist.

We considered it necessary to evaluate the residual anti-Xa effect of apixaban for the estimation of perioperative bleeding and potential need for transfusion; therefore, we implemented the use of ClotPro in combination with the conventional blood tests performed at our hospital’s central laboratory. ClotPro uses RVV as the reagent to activate coagulation. Because RVV specifically cleaves factor X to Xa, clotting time (CT) is prolonged by anti-Xa drugs, such as apixaban, in a dose-dependent manner [3, 4]. We examined blood cell count, PT, APTT, and plasma fibrinogen level at four time points: after anesthesia induction, at the end of surgery, on postoperative day (POD) 1, and on POD 6. We simultaneously performed the ClotPro assay, an ecarin chromogenic assay (ECA), to detect the effect of direct thrombin inhibitors such as dabigatran [5], and a fibrinogen concentration assay. Blood samples for outsourced chromogenic anti-Xa assay were collected at the same time points.

**Fig. 1:**
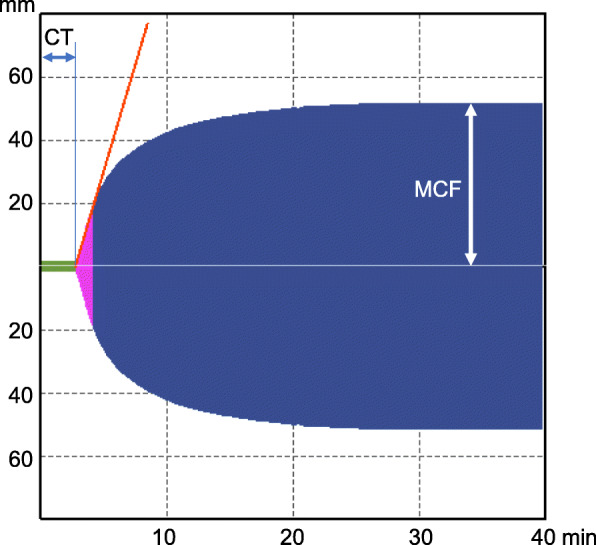
A waveform of the RVV-test after anesthesia induction. Clotting time (CT) is prolonged to 167 s (normal range 48–77 s). CT is a time duration between initiation of the test and the time when clot firmness reaches 20 mm. CT indicates the ability of thrombin generation and is prolonged by anticoagulants such as heparin. MCF represents maximum clot firmness which is defined by plasma fibrinogen level and platelet number and function

Anesthesia was induced with propofol and rocuronium, and was maintained with desflurane and continuous infusion of remifentanil. The results of coagulation tests after anesthesia induction indicated delayed thrombin production (Table 1) (Fig. 1). All members of the surgical team recognized the residual effects of apixaban, and we prepared fresh frozen plasma for excessive bleeding. Fortunately, the volume of bleeding was 440 mL, and we did not perform a transfusion. Considering the balance of the risk of postoperative bleeding and the result of the RVV test on the morning of POD 1, which was in the normal range, we resumed apixaban at a decreased dose of 2.5 mg twice daily on POD 1. The CT for the RVV test on POD 6 was prolonged to 93 s, which was shorter than the preoperative value (167 s) (Table 1). Although the patient had minor subconjunctival hemorrhaging after the resumption of apixaban, he was discharged without major bleeding or thrombotic complications.

**Table 1:** Perioperative coagulation tests

Coagulation test	Normal range	After anesthesia induction	At end of surgery	POD 1	POD 6
Fibrinogen (mg/dL)	150–400^a^	219	220	249	399
APTT (s)	24–36^a^	40.3	38.0	38.5	34.7
PT (%)	70–110^a^	42	49	59	69
PT-INR	0.9–1.1^a^	1.7	1.5	1.3	1.2
CT in RVV test (s)	48–77^b^	167	126	73	93
CT in ECA test (s)	68–100^b^	76	77	73	71
Anti-Xa activity (IU/mL)	< 0.1^b^	1.69	1.12	0.06	0.78

*APTT* Activated partial thromboplastin time, *CT* Clotting time, *ECA* Ecarin, *INR* International normalized ratio, *POD* Postoperative day, *PT* Prothrombin time, *RVV* Russell viper venom, *Xa* Activated factor X

^a^Based on the institutional reference values

^b^Based on the manufacturer’s reference values

## Discussion

Major bleeding owing to the residual effect of apixaban was the major concern in the present case; however, the volume of blood loss was 440 mL, and red blood cell transfusion was not required perioperatively. Although a percutaneous technique for posterior spine fusion might contribute to the moderate amount of bleeding, the perioperative bleeding risk for spine surgery is usually recognized as high [6]. The principles of perioperative management of antithrombotic therapy are interruption of antithrombotic drugs before surgery and prompt resumption after confirmation of surgical hemostasis. The European Heart Rhythm Association recommends 48 h or longer for the interruption period for direct oral anticoagulants before high-bleeding-risk interventions including spine surgery [7], although the timing of resumption depends on the balance between the risks of thrombosis and postprocedural bleeding in each patient. The present patient continued apixaban until the night before surgery; the duration between the last apixaban dose and the first blood sample collection in the operating room (after anesthesia induction) was 15 h. Given the plasma terminal half-life of apixaban of approximately 10 h [8], we expected that the apixaban plasma concentration would be at approximately trough level at the start of surgery.

The values for PT and APTT after anesthesia induction were slightly prolonged, consistent with the increased plasma concentration of apixaban [9]. However, the relation between the plasma concentration of apixaban and PT or APTT is poor; the slopes of the regression lines are relatively small, indicating that PT and APTT are not particularly sensitive to apixaban [1, 10]. The chromogenic anti-Xa assay is a functional test for anti-Xa activity, which has been reported to correlate well with plasma apixaban concentration [10, 11]. The values of plasma anti-Xa activity after anesthesia induction and at the end of surgery were 1.69 IU/mL and 1.12 IU/mL, respectively, in the present case (Table 1). It has been reported that the trough level of apixaban in patients with a dosage of 5 mg twice daily is 1.0 IU/mL [12–14]. Hence, the antithrombotic property of apixaban was considered to be in the therapeutic range during the present surgery. It might be desirable to perform a chromogenic anti-Xa assay to estimate perioperative bleeding risk owing to apixaban; however, it is usually feasible only in a specialized laboratory and has a long turnaround time. We sent blood samples for anti-Xa assay to an outside institution and obtained results several days later.

**Fig. 2:**
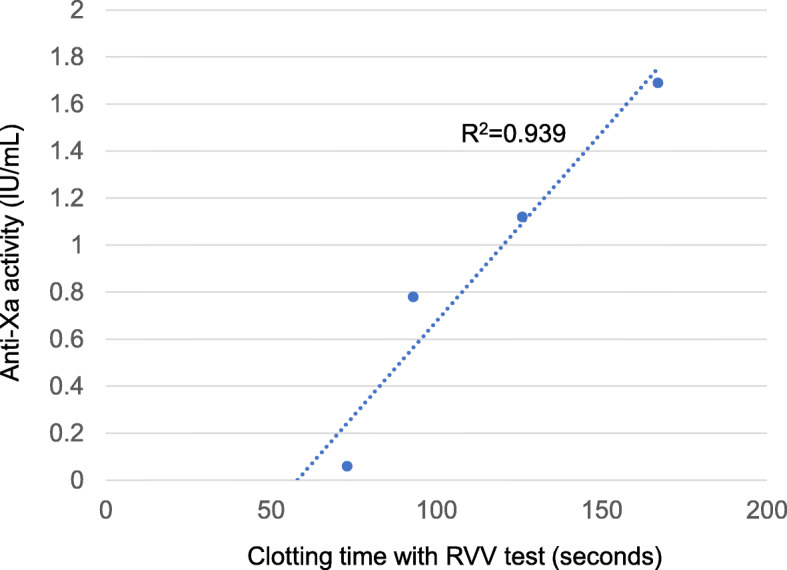
Relation between clotting time with the RVV test and chromogenic anti-Xa activity

We examined the potential of an RVV test performed with ClotPro for evaluation of the antithrombotic effect of apixaban because a point-of-care device with a short turnaround time is suitable for the perioperative period. The RVV test has been developed on the same principle as RVV time (RVVT), which is another functional test for the evaluation of anti-Xa activity; RVVT is an assay for the detection of lupus anticoagulants, but it is also affected by anti-Xa drugs, including apixaban, because RVV specifically cleaves factor X to Xa [3, 4, 15]. As shown in Table 1, the results of the RVV test after anesthesia induction showed prolonged CT, which also showed a proportional linear relation to anti-Xa activity in the therapeutic range of apixaban (Fig. 2). These results might indicate feasibility of the RVV test as a point-of-care assay for evaluation of the anticoagulant effect of apixaban, although an appropriate observational study is required to validate its clinical usefulness.

Because the end point for determination of CT is fibrin clot formation, similar to other viscoelastic tests, prolonged CT with an RVV test might not always reflect anti-Xa activity. Considering the coagulation cascade, it could be also affected by anti-thrombin drugs, such as argatroban or dabigatran, or by hypofibrinogenemia. Thus, we also estimated anti-thrombin activity with the ECA and examined the plasma fibrinogen level [5]. The results were consistently in the normal range during the perioperative period, confirming that the prolongation of CT in the RVV test reflected anti-Xa activity owing to apixaban. Thus, it is reasonable to perform the RVV test in combination with the ECA to specifically evaluate anti-Xa activity.

In conclusion, we described the evaluation of the residual anti-Xa effect of apixaban with an RVV test using ClotPro for a patient who had a minimal interruption period of apixaban perioperatively. Given that continuation of anti-Xa drugs is generally accompanied by increased risks of bleeding and transfusion, the point-of-care evaluation of anti-Xa effect could help physicians prepare for massive bleeding. The short turnaround time for the RVV test using ClotPro might be useful for evaluation of the anti-Xa level induced by apixaban during the perioperative period. A future prospective study should elucidate the clinical usefulness of the RVV test.

## Data Availability

Not applicable.

## Abbreviations

APTT: Activated partial thromboplastin time
CT: Clotting time
POD: Postoperative day
PT: Prothrombin time
RVV: Russell’s viper venom
Xa: Activated factor X
